# The pill millipede genus *Rhopalomeris* Verhoeff, 1906 new to the fauna of China, with an integrative description of a new species from Guangdong Province (Diplopoda, Glomerida, Glomeridae)

**DOI:** 10.3897/zookeys.1280.190182

**Published:** 2026-05-20

**Authors:** Zongji Fan, Hengjie Huang, Zekun Zhang, Li Zhang, Weixin Liu

**Affiliations:** 1 South China Botanical Garden, Chinese Academy of Sciences, Guangzhou 510650, China College of Resources and Environment, Zhongkai University of Agriculture and Engineering Guangzhou China; 2 Administration of Dinghushan National Nature Reserve, Zhaoqing 526070, China Administration of Dinghushan National Nature Reserve Zhaoqing China; 3 Department of Entomology, College of Plant Protection, South China Agricultural University, Guangzhou 510642, China South China Botanical Garden, Chinese Academy of Sciences Guangzhou China; 4 College of Resources and Environment, Zhongkai University of Agriculture and Engineering, Guangzhou 510225, China Department of Entomology, College of Plant Protection, South China Agricultural University Guangzhou China; 5 School of Ecology, Sun Yat-sen University, Shenzhen 518107, China School of Ecology, Sun Yat-sen University Shenzhen China

**Keywords:** China, DNA barcoding, new record, new species, phylogeny, taxonomy

## Abstract

The order Glomerida (pill millipedes) represents a group of terrestrial invertebrates of high ecological and taxonomic significance, yet its diversity and distribution in China remain poorly documented. Employing an integrative taxonomic approach that combines traditional morphological examination with COI gene sequence analysis, this study describes a new species of the genus *Rhopalomeris*, *R. dinghushan***sp. nov**., collected from Dinghushan National Nature Reserve, Guangdong Province. This discovery represents the first formal record of the genus *Rhopalomeris* from China, thereby filling in a major distributional gap for this endemic Oriental genus and extending its known range north of Indochina to southern China. Phylogenetic analyses based on COI sequences, using maximum likelihood and Bayesian inference, reveal that the genus as currently circumscribed seems to be polyphyletic. The new species, *R. dinghushan***sp. nov**., forms a well-supported clade together with *Peplomeris magna*, challenging the current generic delimitation and highlighting the need for a comprehensive revision of the genus.

## Introduction

China is renowned for its rich ecosystems and exceptional species diversity, ranking among the world’s most biodiverse countries (Li and Pimm 2020; Wang et al. 2020). To protect this valuable resource, China has established over 11,800 protected areas at various levels, including national parks, nature reserves, and natural parks, among which 36 sites have been inscribed into the UNESCO World Network of Biosphere Reserves (UNESCO 2025). Among these, Dinghushan National Nature Reserve holds a particularly significant status, for it is not only the first nature reserve ever established in China, but also one of the first sites in the country to join the UNESCO “Man and the Biosphere Programme” (UNESCO 2025). Located in Dinghu District, Zhaoqing City, Guangdong Province, the reserve covers an area of 1133 hm^2^ and experiences a southern subtropical monsoon humid climate, characterized by abundant heat, ample rainfall, and distinct dry and wet seasons (Kong et al. 1993). The reserve supports a diverse array of vegetation types, with its primary conservation objective being the preservation of southern subtropical zonal forest vegetation and its associated biodiversity. To date, various groups—including higher plants, macrofungi, and birds—have been systematically documented within the reserve (Cao et al. 2002; Xiao et al. 2014; Huang 2015; Fan et al. 2023, 2024). However, knowledge of the millipede fauna (Diplopoda) in this reserve remains extremely scarce, with their species composition and distribution patterns still being largely unknown (Golovatch and Liu 2020).

Millipedes (class Diplopoda) represent an ancient, diverse, and globally distributed group of terrestrial invertebrates. Due to their poor dispersal abilities, they are highly dependent on forest and woodland habitats, making them sensitive indicators of habitat integrity and environmental change (Liu et al. 2017). As key detritivores in forest ecosystems, millipedes play indispensable roles in nutrient cycling and soil formation processes (Golovatch and Kime 2009). To date, about 400 species of millipedes, belonging to 71 genera, 26 families, and 11 orders, have been recorded from China (Golovatch and Liu 2020; Zhao et al. 2022; Chen et al. 2024). Within this fauna, the order Glomerida is predominantly distributed in the Holarctic region, and it currently comprises only three families: Glomeridae Leach, 1816, Protoglomeridae Brölemann, 1913, and Glomeridellidae Cook, 1896 (Enghoff et al. 2015). In China, the Glomerida remains a poorly studied group, with only 38 species documented to date, all belonging to two genera: 37 species of *Hyleoglomeris* (Glomeridae) and one species of *Tonkinomeris* (Glomeridellidae) (Liu and Golovatch 2020; Zhang 2025).

During recent biodiversity surveys conducted in Dinghushan National Nature Reserve, a number of millipede specimens were collected. Preliminary morphological examination revealed these specimens to belong to *Rhopalomeris* Verhoeff, 1906 (Glomeridae), a genus primarily diagnosed by the presence of numerous sensory cones on the 8^th^ antennomere (Golovatch 2017; Nguyen et al. 2019, 2021; Likhitrakarn et al. 2024; Sapparojpattana et al. 2025, 2026; Golovatch and Korotayeva 2026). *Rhopalomeris* is endemic to the Oriental region, currently comprising only fourteen described species distributed across Indochina, ranging from northern Vietnam in the north to Peninsular Malaysia in the south, and west to Myanmar (Golovatch and Korotayeva 2026; Sapparojpattana et al. 2026).

Employing an integrative taxonomic approach that combines traditional morphological characterization with DNA barcoding of the mitochondrial cytochrome *c* oxidase subunit I (COI) gene, we describe a new species of *Rhopalomeris* from China. Additionally, genetic distance analyses and phylogenetic reconstruction are conducted to clarify the systematic position of the new species within the genus and to explore its relationships with congeners. The present discovery marks the first formal record of this genus from China, thereby filling a significant distributional gap in its known range.

## Material and methods

### Morphological study

Sampling was accomplished by hand during a field survey conducted at 3:00 AM. Specimens were preserved in 75% ethanol. All type material is deposited in the Zoological Collection of the South China Agricultural University (SCAU), Guangzhou, China. The pictures of the live animals were taken with a Canon EOS R5 camera. Morphological studies were examined using a Leica S8 APO stereo microscope. Photographs were taken with a Keyence VHX-5000 digital microscope. Line drawings were prepared with a Zeiss Axioskop40 microscope with an attached camera lucida. All images were further edited and arranged in plates in Adobe Photoshop CS6.

The terminology used to describe the morphological structures is consistent with that applied in the most recent publications (Liu and Golovatch 2020; Likhitrakarn et al. 2024; Golovatch and Korotayeva 2026).

### DNA extraction and sequencing

Genomic DNA was extracted from muscle tissue using a TIANamp Micro DNA kit (DP316) following the manufacturer’s extraction protocol. The mitochondrial cytochrome *c* oxidase subunit I (COI) gene was amplified using the degenerate primer pair HCO2198-JJ/LCO1490-JJ (Astrin and Stüben 2008). The PCR amplification was performed using a T100™ thermal cycler with a final reaction volume of 25 μL. Sanger sequencing results were edited and assembled using the SeqMan module of Lasergene, and the identities confirmed with BLAST searches (Altschul et al. 1997). Three new nucleotide sequences of COI in this study have been deposited in GenBank. In addition, 38 sequences were downloaded from GenBank. All species analyzed, along with voucher numbers and GenBank accession numbers, are listed in Table 1.

**Table 1.** List of the species used for molecular phylogenetic analyses and their relevant information. Specimens marked by an asterisk have been newly sequenced. Abbreviations: CUMZ = Museum of Zoology, Chulalongkorn University, Bangkok, Thailand; FMNH = Field Museum of Natural History, Chicago, USA; IEBR = Institute of Ecology and Biological Resources, Hanoi, Vietnam; MUMNH = Mahidol University Museum of Natural History, Thailand; SCAU = South China Agricultural University, Guangzhou, China; ZFMK = Zoological Research Museum Koenig, Bonn, Germany.

No.	Species	Locality	Voucher numbers	GenBank accession numbers COI
(1)	*Rhopalomeris carnifex*	Myanmar	CUMZ-GLO016-1	PQ219547
(2)	*Rhopalomeris carnifex*	Myanmar	CUMZ-GLO016-2	PQ219548
(3)	*Rhopalomeris carnifex*	Thailand	MUMNH-GLO066-1	PX220330
(4)	*Rhopalomeris carnifex*	Thailand	MUMNH-GLO104-2	PX220335
(5)	*Rhopalomeris carnifex*	Thailand	MUMNH-GLO138-1	PX220343
(6)	*Rhopalomeris carnifex*	Thailand	MUMNH-GLO157-1	PX220344
(7)	*Rhopalomeris dinghushan* sp. nov.*	China	SCAURD1H	PZ310326
(8)	*Rhopalomeris dinghushan* sp. nov.*	China	SCAURD2M	PZ310327
(9)	*Rhopalomeris dinghushan* sp. nov.*	China	SCAURD1F	PZ310328
(10)	*Rhopalomeris dulcia*	Thailand	MUMNH-GLO188-1	PX220356
(11)	*Rhopalomeris dulcia*	Thailand	MUMNH-GLO188-2	PX220357
(12)	*Rhopalomeris lentiginosa*	Thailand	MUMNH-GLO042-1	PX220353
(13)	*Rhopalomeris lentiginosa*	Thailand	MUMNH-GLO042-2	PX220354
(14)	*Rhopalomeris muka*	Thailand	MUMNH-GLO102-1	PX220349
(15)	*Rhopalomeris muka*	Thailand	MUMNH-GLO102-2	PX220350
(16)	*Rhopalomeris nagao*	Vietnam	IEBR-852	MT749411
(17)	*Rhopalomeris nagao*	Vietnam	IEBR-854	MT749392
(18)	*Rhopalomeris nigroflava*	Myanmar	CUMZ-GLO093-1	PQ219549
(19)	*Rhopalomeris nigroflava*	Myanmar	CUMZ-GLO093-2	PQ219550
(20)	*Rhopalomeris punctata*	Thailand	MUMNH-GLO056-1	PX220359
(21)	*Rhopalomeris punctata*	Thailand	MUMNH-GLO056-3	PX220360
(22)	*Rhopalomeris sauda*	Vietnam	IEBR-801	MT749398
(23)	*Rhopalomeris sauda*	Vietnam	IEBR-706	MT749400
(24)	*Rhopalomeris sauda*	Vietnam	IEBR-654	MT749401
(25)	*Rhopalomeris sauda*	Vietnam	IEBR-533	MT749404
(26)	*Rhopalomeris sirindhornae*	Thailand	MUMNH-GLO189-1	PV176402
(27)	*Rhopalomeris sirindhornae*	Thailand	MUMNH-GLO235-1	PV176403
(28)	*Rhopalomeris verhoeffi*	Thailand	MUMNH-GLO018-2	PX220370
(29)	*Rhopalomeris verhoeffi*	Thailand	MUMNH-GLO081-1	PX220377
(30)	*Rhopalomeris verhoeffi*	Thailand	MUMNH-GLO154-1	PX220382
(31)	*Rhopalomeris verhoeffi*	Thailand	MUMNH-GLO198-1	PX220387
(32)	*Rhopalomeris verhoeffi*	Thailand	MUMNH-GLO201-1	PX220391
(33)	*Hyleoglomeris hongkhraiensis*	Thailand	MUMNH-GLO029-2	PP493232
(34)	*Hyperglomeris simplex*	Vietnam	IEBR-605	MT749403
(35)	*Hyperglomeris simplex*	Vietnam	FMNH-SVE-102	MT749410
(36)	*Peplomeris magna*	Vietnam	IEBR-677	MT749405
(37)	*Peplomeris magna*	Vietnam	IEBR-656	MT749408
(38)	*Tonkinomeris huzhengkuni*	China	SCAUTY01	MT522013
(39)	*Tonkinomeris napoensis*	Vietnam	IEBR-804b	MT749396
(40)	*Tonkinomeris napoensis*	Vietnam	IEBR-804a	MT749397
(41)	*Glomeris marginata*	Europe	ZFMK MYR009	FJ409909

### Sequence alignment and genetic distance

The sequences were aligned using Clustal W and edited in BioEdit (Hall 1999). The final aligned dataset included 41 COI sequences with 621 positions. The genetic distance analyses were conducted in MEGA-X (Kumar et al. 2018) using the Kimura 2-parameter model (Kimura 1980). Codon positions included were 1^st^+2^nd^+3^rd^. All ambiguous positions were removed for each sequence pair (pairwise deletion option).

### Phylogenetic analyses

Phylogenetic analyses were conducted on the PhyloSuite v. 1.2.2 platform (Zhang et al. 2020). COI sequences were aligned using codon-aware MACSE software. Maximum likelihood (ML) inference was performed using IQ-TREE for 1000 standard bootstraps. Bayesian inference (BI) analysis was implemented using MrBayes v. 3.2.6 under the partition model (2 parallel runs; 2,000,000 generations), with the initial 25% of the sampled data discarded as burn-in. The resulting phylogenetic trees were visualized and edited with FigTree v. 1.4.4.

## Taxonomy

### Order Glomerida Brandt, 1833

**Family Glomeridae Leach, 1816**

#### Genus Rhopalomeris

Taxon classificationAnimaliaGlomeridaGlomeridae

Verhoeff, 1906

68F3D07E-0579-588C-984B-A5E7AA30C460

##### Type species.

*Rhopalomeris carnifex* (Pocock, 1889).

#### Rhopalomeris dinghushan sp. nov.

Taxon classificationAnimaliaGlomeridaGlomeridae

73DB53FF-0012-598A-BAED-79155E07A5FB

https://zoobank.org/2E7B6503-FF87-4F63-8E51-341A6CBB8519

**Figure 1.**
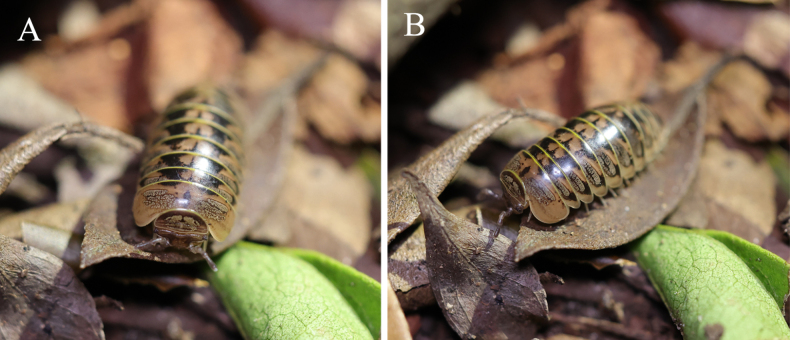
*Rhopalomeris dinghushan* sp. nov. in nature, photos by Zongji Fan.

**Figure 2.**
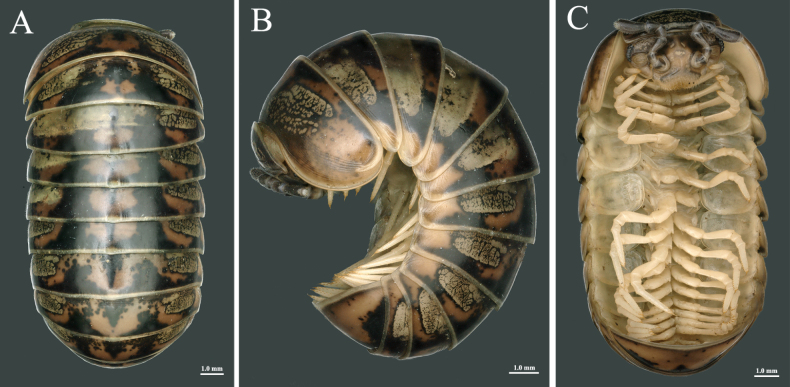
Habitus of *Rhopalomeris dinghushan* sp. nov. **A–C**. Body of holotype, dorsal, lateral and ventral views, respectively.

**Figure 3.**
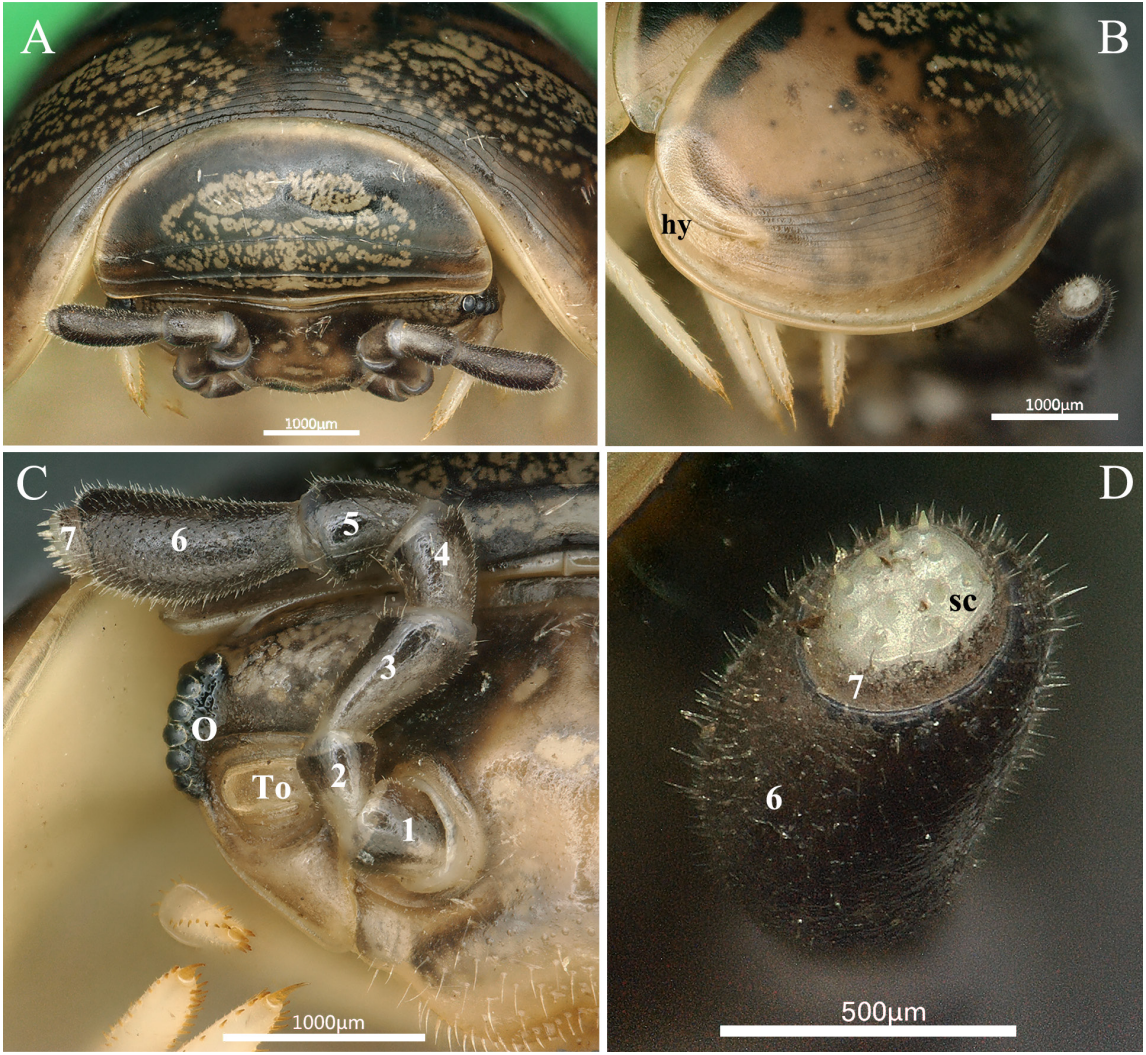
*Rhopalomeris dinghushan* sp. nov., holotype. **A**. Frontal side of head and collum; **B**. Right side of head; **C**. Right side of thoracic shield; **D**. Apical disc with sensory cones. Abbreviations: **hy** hyposchism field, **O** ommatidia, **sc** sensory cones, **To** Tömösváry’s organ. Arabic numbers refer to number of antennomere.

**Figure 4.**
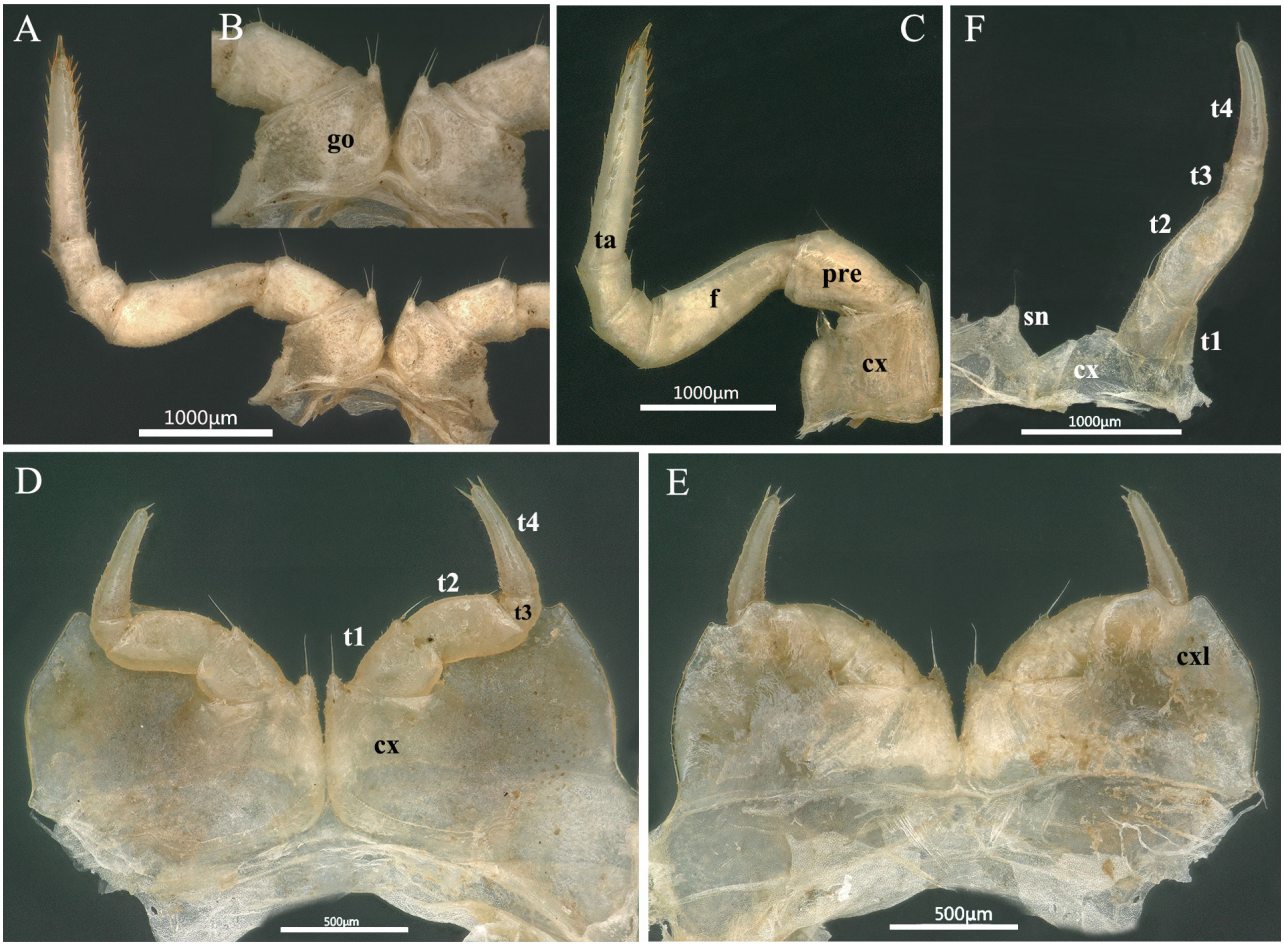
*Rhopalomeris dinghushan* sp. nov., holotype. **A**. Second leg; **B**. Coxa of second leg with gonopore; **C**. Right leg of 9^th^ pair; **D**. Legs 17, anterior view; **E**. Legs 17, posterior view; **F**. Leg 18, anterior view. Abbreviations: **cx** coxa, **cxl** coxal lobe, **f** femur, **go** gonopore, **pre** prefemur, **sn** syncoxital notch, **ta** tarsus. **t1–t4** telopoditomeres 1–4.

**Figure 5.**
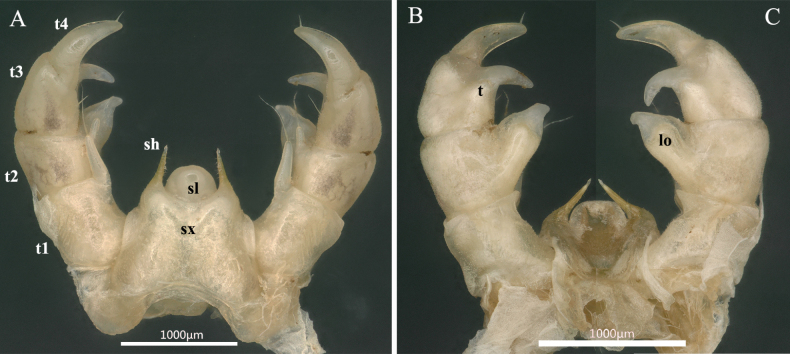
*Rhopalomeris dinghushan* sp. nov., holotype. **A**. Telopods, anterior view; **B**. Telopods, posterior view. Abbreviations: **lo** lobe, **sh** syncoxital horn, **sl** syncoxital lobe, **sx** syncoxite, **t** tubercle, **t1–t4** telopoditomeres 1–4 (= prefemur, femur, tibia and tarsus, respectively).

**Figure 6.**
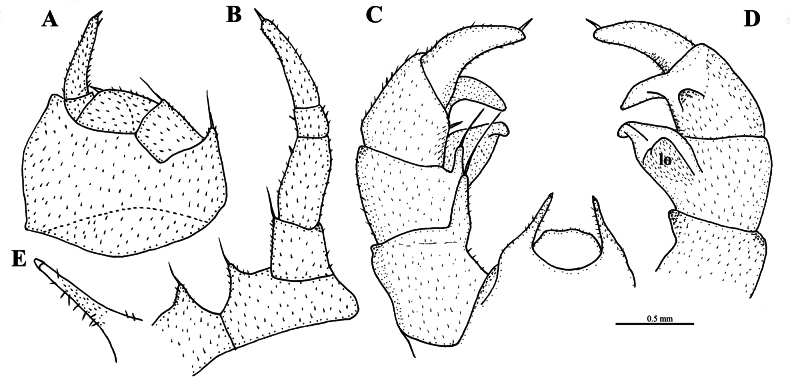
*Rhopalomeris dinghushan* sp. nov., holotype. **A**. Leg 17, anterior view; **B**. Leg 18, posterior view; **C**. Telopod, anterior view; **D**. Telopod, posterior view; **E**. Tip of syncoxital lobe. Abbreviations: **lo** lobe. Scale bar: 1.0 mm (**E**).

Figs 1, 2, 3, 4, 5, 6

##### Type material.

***Holotype***: ♂ (SCAU), China, Guangdong Province, Zhaoqing City, Dinghushan National Nature Reserve, 23.175936°N, 112.517647°E, 708 m, 14.IX.2025, leg. Zongji Fan, Jun Yang, Cuili Chen, Wenbo Bian. ***Paratypes***: 1♂, 1♀ (SCAU), same data as holotype.

##### Diagnosis.

Differs from other species of *Rhopalomeris* by the following combination of characters: (1) body relatively large, length 15.0–21.0 mm (Fig. 2); (2) body blackish-brown, with distinctive yellowish-brown, marbled, paramedian spots (Fig. 2); (3) thoracic shield with about 12 superficial transverse striae (Fig. 3B); (4) male coxae 4–16 each with a triangular apicolateral process (Fig. 4C); and (5) median syncoxital lobe of telopods distinctly lower than lateral horns, each horn with a small apical lobule (Fig. 5).

##### Description.

Based on type specimens. Body length of holotype ca 16.0 mm, maximum width 8.5 mm. Body length of paratypes ca 15.0 mm (male) and 21.0 mm (female), maximum width 7.0 mm (male) and 10.0 mm (female).

***Coloration*** vivid (Figs 1, 2), generally blackish-brown, with contrasting yellowish-brown paramedian spots. Head and antennae largely dark brown; labrum and Tömösváry’s organs lighter, grey-yellowish. Frons with three small, irregular, pale yellow patches arranged in a triangle, and a pair of yellowish-brown, marbled spots above Tömösváry’s organs. Ommatidia greyish-black. Dorsal pattern marbled yellowish-brown: collum with a large, transverse-oval, central spot; thoracic shield and tergites 3–11 each with a pair of similar, transverse-oval, lateral spots, and an irregular, pure brownish, median spot, the latter spot often extending close to lateral spots. Anal shield (= pygidium) mostly brownish except for blackish-brown on both lateral sides and in the middle near frontal margin. Venter and legs light yellowish.

***Head*** (Fig. 2C) densely setose on labrum and clypeus. Ommatidia (Fig. 3C) at least 7+1 in males, but left and right eyes asymmetrical in female, with 14+1/10+1 ommatidia, lenses rather convex. Tömösváry’s organ (Fig. 3C) transverse-oval, parallel to body, slightly wider than long. Antennae of moderate length, all antennomeres densely pubescent. Length ratios of antennomeres: 7 < 4 < 1 = 2 = 5 < 3 < 6, antennomere 6 ca 2.1 times as long as wide (Fig. 3C). Left and right apical cones asymmetrical, with ca 9/12 (holotype) and 7–9/6–8 (paratypes) sensory cones (Fig. 3D).

***Collum*** with two complete transverse striae (Fig. 3A).

***Thoracic shield*** (Figs 2B, 3B) with a narrow hyposchism failing to reach the caudal tergal margin; about 12 superficial transverse striae laterally and dorsolaterally, three or four confused and incomplete; four starting below, one level to, seven above the schism; mid-dorsal region with at least five additional, incomplete, confused striolae.

***Tergites*** (Fig. 2A, B) smooth and shining, paratergites 3 and 4 with three superficial striae, paratergites 4–11 with at least five striae.

***Pygidium*** (Fig. 2B) slightly concave medially at caudal margin in both sexes.

***Legs*** (Fig. 2C) long and slender. All podomeres densely setose, setae being short. Coxae 1–16 each with a distinct, well-rounded apicomesal projection and a long seta, except for coxa 2 occasionally with two setae (Fig. 4A, B). Coxae 4–16 each with a larger, triangular, apicolateral process (Fig. 4C). Tarsi 1–16 each with two rows of 7–9+7+9 dorsal and 8–12+8–12 ventral spines (Fig. 4A, C). In leg-pair 9, femora ca 2.5 times, tarsi 5.5 times as long as wide.

***Male sexual characters***. Gonopore (Fig. 4B) small, oval, with a few short setae around. Leg 17 (Figs 4D, 4E, 6A) strongly reduced. Each coxa with a small, rounded, apicomesal, setigerous projection and a very large, high, irregular, membranous, outer lobe; telopodite 4-segmented; telopoditomere 1 with a similar, but very small apicomesal, setigerous projection; telopoditomere 2 subrectangular; telopoditomere 4 with one apical and one or two subapical spines. Leg 18 (Fig. 4F, 6B) densely micropilose, with a deep, sharp, arcuated, syncoxital notch. Coxa with a small, rounded, apicomesal, setigerous projection. Telopodite 4-segmented. Telopoditomere 1 subrectangular, with an extremely long apicomesal seta. Telopoditomere 2 about two times as long as wide. Telopoditomere 3 smallest, telopoditomere 4 longest, with an apical spine.

***Telopods*** (Figs 5A–C, 6C–E) with a large, roundish, sparsely setose, median, syncoxital lobe flanked by very high setose horns; each horn with a small lobule apically. Telopodite 4-segmented: telopoditomere 1 squarish, micropapillate laterally, with a very long, erect, digitiform, frontomesal trichostele. Telopoditomere 2 with a similar, but smaller frontomesal trichostele, inclined at ca 30°. Caudomedial process of telopoditomere 2 prominent, posteriorly with a rounded lobe covered with short setae, tapering narrowly distally, with a small apical lobe in anterior view. Telopoditomere 3 with a frontomesal seta; caudomesal process evident, recurved; an indistinct, papillate tubercle at base visible in posterior view. Telopoditomere 4 slightly sigmoid, tapering narrowly distally, with a subapical seta.

##### Etymology.

The species is named after its type locality–Dinghu Mountain, the first nature reserve in China. “Dinghushan” in Chinese “鼎湖山”, a noun in apposition.

##### Remarks.

The new species was collected in the core area of Dinghushan National Nature Reserve, under a montane evergreen broad-leaved forest. The dominant plants are *Rapanea neriifoli*a, *Rhododendron henryi* and *Neolitsea chuii*, with accompanying species including *Itea chinensis*, *Diospyros morrisiana*, *Rhodomyrtus tomentosa* and *Enkianthus quinqueflorus*.

### Phylogenetic analysis

#### Genetic distance

The Kimura 2-parameter (K2P) genetic distances of the mitochondrial COI gene are summarized in the distance matrix (Table 2). The distance between *Rhopalomeris* and other glomeridan genera ranged from 12.1% to 18.9%. Among the *Rhopalomeris* species in our dataset, the interspecific genetic distances varied from 8.2% (between *R. lentiginosa* and *R. nigroflava*) to 18.6% (between *R. carnifex* and *R. sauda*). Intraspecific distances between conspecific individuals were 1.6–7.5% in *R. carnifex*, 5.7% in *R. nagao*, 5.9–9.0% in *R. sauda*, 2.0–5.4% in *R. verhoeffi*.

**Table 2.** The K2P genetic distance of the COI gene as calculated by MEGA-X.

Specimens	(1)	(2)	(3)	(4)	(5)	(6)	(7)	(8)	(9)	(10)	(11)	(12)	(13)	(14)	(15)	(16)	(17)	(18)	(19)	(20)	(21)	(22)	(23)	(24)	(25)	(26)	(27)	(28)	(29)	(30)	(31)	(32)	(33)	(34)	(35)	(36)	(37)	(38)	(39)	(40)
(1) *R. carnifex*PQ219547																																								
(2) *R. carnifex*PQ219548	0.016																																							
(3) *R. carnifex*PX220330	0.033	0.050																																						
(4) *R. carnifex*PX220335	0.057	0.075	0.054																																					
(5) *R. carnifex*PX220343	0.042	0.054	0.028	0.059																																				
(6) *R. carnifex*PX220344	0.037	0.050	0.016	0.056	0.028																																			
(7) *R. dinghushan* sp. nov. SCAURD1H	**0.138**	**0.152**	**0.143**	**0.136**	**0.147**	**0.144**																																		
(8) *R. dinghushan* sp. nov. SCAURD2M	**0.138**	**0.152**	**0.143**	**0.136**	**0.147**	**0.144**	**0.000**																																	
(9) *R. dinghushan* sp. nov. SCAURD1F	**0.138**	**0.152**	**0.143**	**0.136**	**0.147**	**0.144**	**0.000**	**0.000**																																
(10) *R. dulcia*PX220356	0.116	0.133	0.114	0.115	0.108	0.120	**0.145**	**0.145**	**0.145**																															
(11) *R. dulcia*PX220357	0.116	0.133	0.114	0.115	0.108	0.120	**0.145**	**0.145**	**0.145**	0.000																														
(12) *R. lentiginosa*PX220353	0.099	0.117	0.095	0.099	0.104	0.099	**0.148**	**0.148**	**0.148**	0.110	0.110																													
(13) *R. lentiginosa*PX220354	0.099	0.117	0.095	0.099	0.104	0.099	**0.148**	**0.148**	**0.148**	0.110	0.110	0.000																												
(14) *R. muka*PX220349	0.110	0.125	0.120	0.101	0.116	0.122	**0.128**	**0.128**	**0.128**	0.097	0.097	0.090	0.090																											
(15) *R. muka*PX220350	0.112	0.127	0.118	0.099	0.114	0.120	**0.128**	**0.128**	**0.128**	0.095	0.095	0.088	0.088	0.005																										
(16) *R. nagao*MT749411	0.134	0.148	0.129	0.134	0.126	0.128	**0.121**	**0.121**	**0.121**	0.137	0.137	0.126	0.126	0.142	0.140																									
(17) *R. nagao*MT749392	0.134	0.148	0.136	0.130	0.136	0.138	**0.132**	**0.132**	**0.132**	0.132	0.132	0.127	0.127	0.128	0.128	0.057																								
(18) *R. nigroflava*PQ219549	0.102	0.117	0.110	0.114	0.117	0.108	**0.144**	**0.144**	**0.144**	0.116	0.116	0.082	0.082	0.100	0.099	0.121	0.124																							
(19) *R. nigroflava*PQ219550	0.102	0.117	0.110	0.114	0.117	0.108	**0.144**	**0.144**	**0.144**	0.116	0.116	0.082	0.082	0.100	0.099	0.121	0.124	0.000																						
(20) *R. punctata*PX220359	0.126	0.145	0.131	0.133	0.137	0.139	**0.144**	**0.144**	**0.144**	0.106	0.106	0.109	0.109	0.108	0.110	0.131	0.133	0.112	0.112																					
(21) *R. punctata*PX220360	0.126	0.145	0.131	0.133	0.137	0.139	**0.144**	**0.144**	**0.144**	0.106	0.106	0.109	0.109	0.108	0.110	0.131	0.133	0.112	0.112	0.000																				
(22) *R. sauda*MT749398	0.137	0.154	0.131	0.133	0.123	0.136	**0.142**	**0.142**	**0.142**	0.133	0.133	0.137	0.137	0.134	0.136	0.121	0.139	0.135	0.135	0.133	0.133																			
(23) *R. sauda*MT749400	0.161	0.175	0.155	0.151	0.140	0.148	**0.156**	**0.156**	**0.156**	0.145	0.145	0.131	0.131	0.130	0.128	0.127	0.125	0.137	0.137	0.153	0.153	0.083																		
(24) R. saudaMT749401	0.144	0.162	0.142	0.143	0.140	0.142	**0.144**	**0.144**	**0.144**	0.129	0.129	0.137	0.137	0.115	0.121	0.135	0.134	0.146	0.146	0.127	0.127	0.068	0.090																	
(25) *R. sauda*MT749404	0.168	0.186	0.157	0.159	0.147	0.157	**0.146**	**0.146**	**0.146**	0.139	0.139	0.139	0.139	0.146	0.144	0.133	0.131	0.141	0.141	0.143	0.143	0.070	0.059	0.079																
(26) *R. sirindhornae*PV176402	0.105	0.121	0.101	0.099	0.097	0.101	**0.144**	**0.144**	**0.144**	0.107	0.107	0.118	0.118	0.118	0.116	0.130	0.146	0.127	0.127	0.129	0.129	0.137	0.147	0.137	0.143															
(27) *R. sirindhornae*PV176403	0.103	0.119	0.099	0.097	0.095	0.101	**0.144**	**0.144**	**0.144**	0.107	0.107	0.116	0.116	0.116	0.114	0.132	0.148	0.125	0.125	0.127	0.127	0.137	0.149	0.137	0.145	0.002														
(28) *R. verhoeffi*PX220370	0.118	0.137	0.112	0.118	0.112	0.116	**0.146**	**0.146**	**0.146**	0.099	0.099	0.116	0.116	0.127	0.125	0.134	0.152	0.133	0.133	0.131	0.131	0.136	0.154	0.140	0.150	0.106	0.106													
(29) *R. verhoeffi*PX220377	0.099	0.117	0.106	0.116	0.112	0.106	**0.150**	**0.150**	**0.150**	0.105	0.105	0.122	0.122	0.135	0.137	0.134	0.148	0.123	0.123	0.138	0.138	0.143	0.159	0.142	0.171	0.105	0.105	0.054												
(30) *R. verhoeffi*PX220382	0.122	0.141	0.114	0.120	0.116	0.118	**0.144**	**0.144**	**0.144**	0.107	0.107	0.120	0.120	0.131	0.129	0.138	0.148	0.127	0.127	0.129	0.129	0.136	0.153	0.148	0.161	0.110	0.110	0.045	0.047											
(31) *R. verhoeffi*PX220387	0.120	0.139	0.112	0.120	0.114	0.118	**0.158**	**0.158**	**0.158**	0.099	0.099	0.116	0.116	0.129	0.127	0.154	0.158	0.127	0.127	0.135	0.135	0.138	0.163	0.148	0.167	0.108	0.106	0.033	0.054	0.045										
(32) *R. verhoeffi*PX220391	0.116	0.135	0.118	0.114	0.116	0.122	**0.146**	**0.146**	**0.146**	0.097	0.097	0.110	0.110	0.123	0.121	0.146	0.154	0.125	0.125	0.125	0.125	0.136	0.161	0.136	0.167	0.112	0.112	0.040	0.047	0.049	0.037									
(33) *H. hongkhraiensis*PP493232	0.119	0.128	0.126	0.121	0.126	0.130	**0.132**	**0.132**	**0.132**	0.130	0.130	0.126	0.126	0.113	0.111	0.121	0.115	0.111	0.111	0.119	0.119	0.132	0.144	0.130	0.142	0.128	0.127	0.142	0.146	0.150	0.144	0.140								
(34) *H. simplex*MT749403	0.146	0.162	0.138	0.148	0.142	0.140	**0.140**	**0.140**	**0.140**	0.152	0.152	0.119	0.119	0.134	0.132	0.121	0.125	0.126	0.126	0.139	0.139	0.149	0.143	0.146	0.126	0.154	0.152	0.146	0.162	0.162	0.154	0.150	0.125							
(35) *H. simplex*MT749410	0.150	0.170	0.136	0.150	0.146	0.146	**0.140**	**0.140**	**0.140**	0.154	0.154	0.117	0.117	0.138	0.140	0.127	0.121	0.134	0.134	0.155	0.155	0.135	0.133	0.136	0.129	0.148	0.146	0.144	0.162	0.156	0.144	0.154	0.134	0.052						
(36) *P. magna*MT749405	0.136	0.156	0.134	0.132	0.134	0.140	**0.123**	**0.123**	**0.123**	0.121	0.121	0.128	0.128	0.128	0.126	0.121	0.124	0.136	0.136	0.130	0.130	0.141	0.125	0.133	0.155	0.140	0.140	0.132	0.135	0.137	0.140	0.135	0.140	0.140	0.136					
(37) *P. magna*MT749408	0.140	0.160	0.138	0.136	0.138	0.144	**0.123**	**0.123**	**0.123**	0.125	0.125	0.130	0.130	0.128	0.126	0.121	0.125	0.136	0.136	0.134	0.134	0.143	0.125	0.133	0.155	0.144	0.144	0.136	0.139	0.140	0.144	0.138	0.144	0.140	0.136	0.003				
(38) *T. huzhengkuni*MT522013	0.159	0.177	0.172	0.160	0.155	0.170	**0.158**	**0.158**	**0.158**	0.133	0.133	0.139	0.139	0.149	0.151	0.151	0.161	0.155	0.155	0.125	0.125	0.154	0.166	0.146	0.154	0.136	0.134	0.149	0.164	0.151	0.156	0.151	0.145	0.135	0.145	0.145	0.149			
(39) *T. napoensis*MT749396	0.141	0.159	0.141	0.149	0.139	0.147	**0.146**	**0.146**	**0.146**	0.143	0.143	0.138	0.138	0.144	0.146	0.159	0.151	0.147	0.147	0.154	0.154	0.135	0.141	0.135	0.135	0.138	0.136	0.147	0.147	0.149	0.152	0.155	0.147	0.153	0.141	0.139	0.141	0.155		
(40) *T. napoensis*MT749397	0.153	0.173	0.153	0.161	0.155	0.159	**0.154**	**0.154**	**0.154**	0.159	0.159	0.145	0.145	0.156	0.158	0.167	0.165	0.151	0.151	0.161	0.161	0.146	0.157	0.150	0.151	0.149	0.148	0.156	0.156	0.158	0.162	0.164	0.163	0.159	0.146	0.149	0.151	0.163	0.016	
(41) *G. marginata*FJ409909	0.160	0.176	0.164	0.174	0.156	0.168	**0.174**	**0.174**	**0.174**	0.170	0.170	0.163	0.163	0.161	0.157	0.166	0.168	0.170	0.170	0.159	0.159	0.184	0.189	0.170	0.175	0.173	0.171	0.179	0.174	0.176	0.174	0.178	0.150	0.170	0.191	0.170	0.168	0.165	0.160	0.168

No intraspecific genetic divergence was detected among the examined individuals of the new species, *R. dinghushan* sp. nov., while its interspecific K2P distances from its congeners ranged from 12.1% (compared to *R. nagao*) to 15.8% (compared to *R. verhoeffi*).

#### Phylogenetic relationships

**Figure 7.**
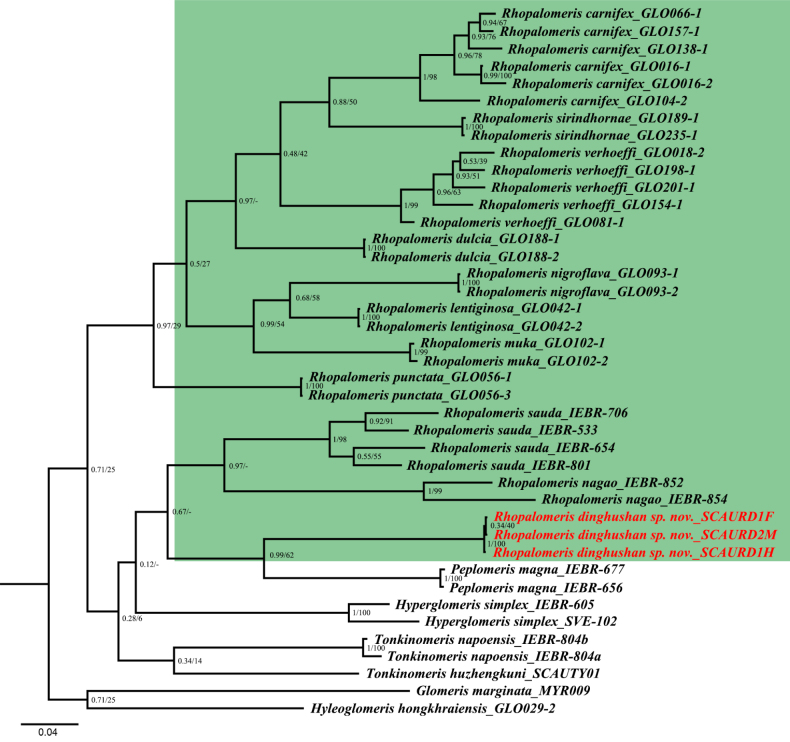
Phylogenetic diagram inferred from Bayesian inference analysis of pill millipedes based on 621 bp of the COI gene. Numbers above branches indicate numbers below branches are bipartition posterior probability (bpp) from BI analysis and Bootstrap Support (BS) values from the ML analysis. Clades of *Rhopalomeris* species are highlighted in green. Scale bar: 0.04 substitutions/site.

The final aligned dataset included 41 sequences, with a total length of 621 bp for the mitochondrial COI gene. The maximum likelihood (ML) and Bayesian inference (BI) analyses revealed largely identical topologies; therefore, only the BI tree is presented here (Fig. 7). All species included in the present study were recovered as distinct, well-supported, monophyletic lineages. However, the higher-level phylogenetic relationships among glomeridan genera were not well resolved and received low nodal support. *Hyleoglomeris hongkhraiensis* and *Glomeris marginata* were placed at the base of the tree, indicating a deep phylogenetic divergence from the remaining taxa.

Notably, the genus *Rhopalomeris* was not recovered as monophyletic, but instead formed two deeply divergent clades. The first clade comprised *R. carnifex*, *R. sirindhornae*, *R. verhoeffi*, *R. dulcia*, *R. nigroflava*, *R. lentiginosa*, *R. muka* and *R. punctata*. However, the sister-group relationships among these species received relatively low support. Within this clade, *R. carnifex* formed a stable, well-supported monophyletic group (bpp = 1.0, BS = 98) and was resolved as the sister taxon to *R. sirindhornae* with moderate support (bpp = 0.88, BS = 50). *Rhopalomeris nigroflava* clustered with *R. lentiginosa* at a moderate level of support (bpp = 0.68, BS = 58) and subsequently grouped with *R. muka* and *R. punctata*. The second clade was further divided into two subclades. Within this clade, *R. sauda* and *R. nagao* formed a sister-group relationship with strongly support in the BI tree (bpp = 0.97). In contrast, the new species *R. dinghushan* sp. nov. was recovered in a separate, well-supported subclade together with *Peplomeris magna* (bpp = 0.99, BS = 62). Closely related genera such as *H. simplex*, *T. napoensis*, and *T. huzhengkuni* are positioned between the genus *Rhopalomeris* and the basal outgroups. The higher-level phylogenetic relationships among these genera remain poorly resolved, and nodal support values are generally low.

## Discussion

In the present study, we describe a new species, *Rhopalomeris dinghushan* sp. nov., collected from Dinghushan National Nature Reserve, Guangdong Province, China. This represents the first formal record of the genus *Rhopalomeris* Verhoeff, 1906 in China, filling in a significant distributional gap for this endemic Oriental genus and extending its known range northward from Indochina to southern China (Likhitrakarn et al. 2024; Sapparojpattana et al. 2025, 2026; Golovatch and Korotayeva 2026). Beyond its biogeographical significance, this discovery also enriches the species diversity of the order Glomerida in China, highlighting the understudied millipede fauna of Dinghushan National Nature Reserve, a site of high ecological and conservation value.

With respect to morphology, Sapparojpattana (2026) provided a taxonomic key to the 13 known species of the genus. *Rhopalomeris dinghushan* sp. nov. can be clearly distinguished from all other congeners by its unique combination of diagnostic characters. Its relatively large body size (15.0–21.0 mm) is considerably larger than that of *R. nigroflava* (5.1–9.7 mm) from Myanmar, *R. lentiginosa* (7.6–11.8 mm) and *R. punctata* (8.3–10.3 mm) from Thailand, and *R. monacha* (12 mm) from Malaysia (Likhitrakarn et al. 2024). The thoracic shield bears approximately 12 superficial transverse striae, a feature that markedly differs from *R. sauda*, which possesses only 2–3 striae. Notably, the distinctive yellowish-brown, marbled paramedian spots against a blackish-brown background form a colour pattern that fails to match any previously described species of *Rhopalomeris*. This pattern also clearly distinguishes the new species from those with red or pale pink markings along the lateral margins of the tergites, including *R. carnifex*, *R. dulcia*, *R. sirindhornae*, and *R. verhoeffi*. While colouration in this genus is known to be highly variable and taxonomically informative (Golovatch 2017; Golovatch and Korotayeva 2026; Sapparojpattana et al. 2026), the unique color pattern of the new species further supports its distinctiveness. Additional reliable diagnostic features include the presence of triangular apicolateral processes on coxae 4–16 and the characteristic morphology of the telopods, both of which are consistent with the genus *Rhopalomeris* but are distinct from other congeners.

Complementing the morphological evidence, genetic distance analyses based on the COI gene provide robust support for the specific distinctness of *R. dinghushan* sp. nov. The Kimura 2-parameter (K2P) interspecific genetic distances between the new species and its congeners range from 12.1% (vs. *R. nagao*) to 15.8% (vs. *R. verhoeffi*), the values that substantially exceed the commonly accepted threshold for species-level differentiation in millipedes and other invertebrates (Hebert et al. 2003; Wesener 2015). These estimates are consistent with the interspecific divergences (10.85–16.13% based on uncorrected p-distances) reported for *Rhopalomeris* species by Likhitrakarn et al. (2024). Furthermore, no intraspecific genetic variation was detected among the three sequenced individuals of *R. dinghushan* sp. nov., further supporting the cohesive nature of this population and confirming that it represents a single, well-delimited species.

Perhaps the most striking result of our phylogenetic analyses is the likely polyphyly of the genus *Rhopalomeris* as currently circumscribed. The genus is resolved into two deeply divergent clades: one comprising eight species, including *R. carnifex* and allied taxa, and the other containing *R. sauda*, *R. nagao*, *R. dinghushan* sp. nov., and *Peplomeris magna*. Notably, *R. dinghushan* sp. nov. is recovered as sister to *P. magna* within the second clade, rather than grouping with other *Rhopalomeris* species—an unexpected finding that raises intriguing questions about character evolution and biogeographical patterns within this group. Morphologically, *Rhopalomeris* and *Peplomeris* are the only genera in the order Glomerida known to possess numerous sensory cones on the 8^th^ antennomere, a shared feature that may reflect a close evolutionary relationship. However, the two genera can be reliably distinguished by key morphological traits: the telopod prefemur bears a trichostele that ranges from rudimentary to long and fully developed in *Rhopalomeris*, whereas it is consistently rudimentary in *Peplomeris*; additionally, *Peplomeris* possesses two paramedian knobs at the caudal margin of the male pygidium (Golovatch 1983), a character absent from *Rhopalomeris*.

Several limitations of this study should be acknowledged. Firstly, our phylogenetic inferences are based solely on a single mitochondrial marker (COI); mitochondrial genes alone may not fully resolve the true phylogenetic relationships among higher-level taxa, as they are prone to introgression and may not reflect nuclear evolutionary histories. Secondly, the genus *Rhopalomeris* currently lacks clear morphological synapomorphies, and its primary diagnostic feature (numerous sensory cones on the 8^th^ antennomere) may represent a plesiomorphic or convergent character, which could explain the polyphyletic topology observed here. Finally, while our taxon sampling includes all *Rhopalomeris* species with available sequence data, it does not encompass all described species of the genus, potentially introducing sampling bias. Future studies integrating multiple nuclear and mitochondrial markers, combined with detailed morphological re-examinations and expanded taxon sampling, will be essential to clarify the systematic position of *Rhopalomeris* and resolve generic boundaries within this ecologically and taxonomically important group.

## Supplementary Material

XML Treatment for Rhopalomeris

XML Treatment for Rhopalomeris dinghushan

## Additional information

### Artificial Intelligence (AI) use

The authors accept full responsibility for the content of the manuscript, including the disclosure of any use of AI.

No AI tools were used in the preparation of this manuscript.

### Author ORCIDs

Z. Fan https://orcid.org/0009-0001-9129-423X

Z. Zhang https://orcid.org/0009-0009-3705-1825

L. Zhang https://orcid.org/0009-0001-1082-5001

W. Liu https://orcid.org/0000-0001-9122-8868

### Data availability

All of the data that support the findings of this study are available in the main text.
